# Supplementary material for: Content-rich biological network constructed by mining PubMed abstracts
Source: BMC Bioinformatics. 2004 Oct 8;5:147. doi: 10.1186/1471-2105-5-147 (PMC528731; doi:10.1186/1471-2105-5-147)
Supplement: Additional File 2 — The original results of the above study (non-essential files are deleted to keep the file size under the limit set by BMC bioinformatics). [file 1471-2105-5-147-S2.bz2 › chilibotAdditionalFile2/dip05/145ID8955331E515/html/RSBV_RSBW.html]

 


 **RSBV** and **RSBW** 
  
Found 25 abstracts in PubMed, retrieved 05.  
 

 What does Google say? 
 PDF only 
| .edu only 

---

**Interactive relationship** (e.g. stimulation, inhibition, etc)

**Neutral relationship**- **RsbW**  is also a protein kinase which can phosphorylate  **RsbV** .  Ref: 12270815 J Bacteriol, 2002
- When cells are stressed,  **RsbW**  binds to unphosphorylated  **RsbV** , produced from the phosphorylated form of  **RsbV**  by two phosphatases RsbU and RsbP which are activated by stress.  Ref: 12270815 J Bacteriol, 2002
- **RsbW** , purified as a dimer, forms complexes with  **RsbV**  and sigma B with different stoichiometries, i.e.,  **RsbW**  2  **RsbV**  2 and  **RsbW**  2 sigma B 1.  Ref: 12270815 J Bacteriol, 2002
- We conclude that the higher affinity of  **RsbW**  for  **RsbV**  than for sigma B, rather than a difference in the concentrations of  **RsbV**  and sigma B, is the driving force that is responsible for the switch of  **RsbW**  to unphosphorylated  **RsbV** .  Ref: 12270815 J Bacteriol, 2002

**Non-interactive relationship** (e.g. studied together, co-existance, homology, etc.)

- The increase was greatest for  **RsbV** , whose concentration reached 13 micro M, while those of  **RsbW**  as a monomer and sigma B reached 11.8 and 4.9 micro M, respectively.  Ref: 12270815 J Bacteriol, 2002
- Nonetheless, an analysis of the complexes by nondenaturing polyacrylamide gel electrophoresis in competition assays suggested that the affinity of  **RsbW**  2 for  **RsbV**  is much higher than that for sigma B.  Ref: 12270815 J Bacteriol, 2002
- As determined by surface plasmon resonance, the dissociation constants of the  **RsbW**   **RsbV**  and  **RsbW**  sigma B interactions were found to be similar 63 and 92 nM, respectively.  Ref: 12270815 J Bacteriol, 2002
- The intracellular concentrations of  **RsbV** ,  **RsbW**  as a monomer, and sigma B measured before stress were similar 1..6, and 0.9 micro M, respectively.  Ref: 12270815 J Bacteriol, 2002
- The operon encoding the general stress transcription factor sigma B and two proteins of its regulatory network,  **RsbV**  and  **RsbW** , was cloned from the gram positive bacterium Bacillus anthracis by PCR amplification of chromosomal DNA with degenerate primers, by inverse PCR, and by direct cloning.  Ref: 10960085 J Bacteriol, 2000
- These two classes of stress are transmitted by separate signaling pathways which converge on the direct regulators of sigma B, the  **RsbV**  anti anti sigma factor and the  **RsbW**  anti sigma factor.  Ref: 11591687 J Bacteriol, 2001
